# High dives and parallel plans: Relationships between medical student elective strategies and residency match outcomes

**DOI:** 10.36834/cmej.53018

**Published:** 2020-07-15

**Authors:** Carol Ann Courneya, Winson Cheung, M. Janette McMillan

**Affiliations:** 1 University of British Columbia, British Columbia, Canada; 2 University of Calgary, Alberta, Canada

## Abstract

**Background:**

Medical students are anxious about not getting a preferred residency position. We described elective patterns of two recent cohorts and examined associated match outcomes.

**Methods:**

We conducted a retrospective review of the final-year electives of all students who participated in the residency match (first iteration) at one school for 2017 and 2018. We categorized elective patterns and associated them with aggregated match outcomes. We examined high-demand/low-supply (HDLS) disciplines separately.

**Results:**

We described three elective patterns: High Dive, Parallel Plan(s), and No Clear Pattern. Many students had High Dive and Parallel Plans patterns; only a few showed No Clear Pattern. Match rates for High Dive and Parallel Plan patterns were high but many students matched to Family and Internal Medicine. When we separated out HDLS predominance, the match rate remained high but a significant number matched to disciplines in which they did not have a majority of electives. Most High Dive and Parallel Plan students who went unmatched did so with HDLS discipline electives.

**Conclusion:**

Many students chose High Dive and Parallel Plan strategies to both high-capacity and HDLS disciplines. Match rates were high for both patterns but students also matched to non-primary disciplines. Back-up planning may reside in the entire application, and not just electives selection.

## Abstract (fr)

**Contexte:**

Les étudiants en médecine sont anxieux à l’idée de ne pas obtenir le poste de résidence souhaité. Nous avons décrit les profils de stages à optionde deux cohortes récentes et examiné les résultats des jumelages associés.

**Méthodes:**

Nous avons mené une évaluation rétrospective des stages à option d’externat senior effectués par l’ensemble des étudiants qui ont participé au jumelage de résidence (premier tour) dans un programme de médecine pour 2017 et 2018. Nous avons classé les profils de stage et les avons associés aux résultats de jumelage agrégés. Nous avons examiné les disciplines à demande élevée et à offre faible (DEOF) séparément.

**Résultats:**

Nous avons décrit trois profils de stages à option : le « grand saut », le plan parallèle B et aucun schéma précis. De nombreux étudiants présentaient des profils de type « grand saut » et plans parallèles. Seuls quelques-uns ne présentaient aucun profil précis. Les taux de jumelage pour les schémas grand saut et plan parallèle étaient élevés, mais de nombreux étudiants étaient jumelés à Médecine familiale et Médecine interne. Quand nous avons séparé la prédominance DEOF, le taux de jumelage restait élevé, mais un nombre important d’étudiants obtenaient un poste dans des disciplines pour lesquelles ils n’avaient pas fait une majorité de stages à option. La plupart des étudiants avec profils « grand saut » et plan parallèle qui n’avaient pas été jumelés avaient fait des stages à option dans des disciplines DEOF.

**Conclusion:**

De nombreux étudiants avaient choisi des stratégies « grand saut » et plan parallèle pour des disciplines à haute capacité ainsi que des disciplines DEOF. Les taux de jumelage étaient élevés pour les deux profils, mais les étudiants se jumelaient également à des disciplines autres que leur premier choix. Une solution de rechange peut se trouver dans l’ensemble du processus, et non pas seulement dans la sélection des stages à option.

## Introduction

In 1985, Dr. Swanson coined the term “preresidency syndrome” to describe a phenomenon where medical students were excessively preoccupied with gaining a position in a graduate medical education program of their choice. At that time, there were about 4000 more training positions than American medical student graduates.^1^^,^^2^ More recently, senior U.S. medical students have been applying to increased numbers of desirable residency programs. This is labeled “Residency Placement Fever.”^3^

Medical students enter the Canadian Residency Match Service (CaRMS) to determine their residency match to a Canadian program/school. In Canada, an expansion of residency positions preceded subsequent medical school position expansion. Now, the medical school positions closely match the number of residency positions, thus we have a diminished ratio of residency positions to the number of Canadian medical students (very close to one-to-one), and an increasing number of unmatched medical students.^4^ This new reality and the media interest in it have fueled student anxiety over the possibility of being unmatched, the consequence of which is entering the second iteration of the match or potentially sitting out a year waiting to enter the match one year later. An exploration of the personal emotional toll of going unmatched has recently been reported by an unmatched student.^5^ In a system where students could perceive themselves to have little control over the process, their selection of electives is one aspect about the match process that they can oversee and change. We defined “pre-CaRMS electives” for this study as senior medical student placements that would provide sufficient time to obtain a letter of reference.

In Canada, elective selection discussions often happen in career advising sessions with faculty and personnel from Student Affairs. During these sessions, students ask about various pre-CaRMS elective strategies and, to date, such discussions have been largely driven by anecdotal data, most of which have been gleaned from the examination of strategies of students who have not matched. In an opinion piece published in the Canadian Medical Association Journal, M. Berchard, a medical student, suggested that being privy to the types of electives of successful applicants would provide some useful insights for future students who are planning for their own senior medical student elective experiences.^6^ We did not find any studies that have examined elective strategies and their relationship to match outcome.

Therefore, we had two purposes for this study:

1. to conduct a retrospective descriptive data analysis of pre-CaRMS electives to describe over-arching elective patterns and, by inference, of the strategies of recent senior medical student students;
2. to describe the aggregate match outcomes associated with these different elective approaches.

## Methods

The UBC MD program consists of two years of foundatonal science integrated with early clinical exposure and clinical skills training. Thrid year consists of rotating or Integrated clerkship that occurs in a hospital setting. Fourth year is largely made up of 2 to 4 week long electives. UBC UGME students have had twenty weeks of pre-CaRMS electives. Students at UBC have been permitted to do a maximum of 12 weeks in a single CaRMS discipline.

The University of British Columbia (UBC) Undergraduate Medical Education (UGME) Student Affairs compiled the list of electives for graduating medical students who entered the first iteration residency matches in 2017 and 2018 (n= 543). We did not subcategorize the electives in any other specific ways or by other parameters, such as location either within British Columbia or out-of-province, supervisor/elective manager, or description of activities and objectives. We restricted our data set to the numbers of weeks spent in each discipline preceding the submission deadline for letters of reference in the CaRMS process. For these years, the last possible opportunity for reference letters was mid-November.

For this study, we defined a successful match outcome as a graduating medical student who obtained a PGY1 position after the first iteration of the CaRMS process. Two of the authors (CAC and WYC) initially individually characterized the combination of electives for each student with descriptions of elective patterns. We discussed these preliminary text descriptors and subsequently collapsed some category descriptors and separated out others. We came to agreement on the final text descriptors. The third author (MJM) reviewed these final text descriptors. All authors agreed on three broad elective pattern categories: High Dive, Parallel Plan(s), and No Clear Pattern. For the High Dive pattern, we inferred the students’ primary discipline of choice on the basis of a selection of the majority of electives in that discipline. For the Parallel Plan(s) pattern, we inferred the students’ one or two primary disciplines of choice on the basis of a selection of the majority of electives in those two disciplines. For the No Clear Pattern category, students had a wide variation of elective choices and thus we could not infer the students’ chosen primary discipline.

We used chi-squared analyses to correlate the match outcome (that is, whether the students matched and to which discipline) with each of the three patterns of pre-CaRMS electives.

For each elective pattern, we calculated the percentage match outcome initially with all disciplines included and then re-calculated the percentage match outcome having removed the high-capacity disciplines of Family Medicine and Internal Medicine. We have called the second group of disciplines (with Family Medicine and Internal Medicine removed) “high-demand/low-supply” (HDLS).

We present these data as percentages with 95% confidence intervals to protect the privacy of individual students.

The University of British Columbia Ethics Board (H17-01005) approved this study.

## Results

We present first the three elective patterns we determined, the percentage of each pattern in the classes, the match outcome associated with each elective pattern, and, finally, elective patterns associated with students that went unmatched.

### Pre-CaRMS elective patterns

We determined three patterns for pre-CaRMS electives:

1. High Dive
2. Parallel Plan(s)
3. No Clear Pattern

***High dive pattern*.** “High Dive” pattern means students had chosen to do 10 or more weeks of electives in a single CaRMS entry discipline. An example of this was 12 weeks of electives in Urology.

***Parallel plan(s) pattern*.** “Parallel Plan(s)” pattern referred to the students who pursued one of three actions: a) six or more weeks in two CaRMS-entry disciplines, or b) one primary discipline for four to six weeks plus a variety of two- or four-week electives that would be considered generally supportive of the primary discipline (e.g., Family Medicine plus Psychiatry, Obstetrics, Internal Medicine, and Radiation Oncology).

***No clear pattern*.** We called the third category “No Clear Pattern” since it consisted of a wide variety of two- or four-week, disparate CaRMS entry-level disciplines (e.g., Urology, Pathology, Public Health, Internal Medicine, Radiation Oncology, and Paediatrics).

Table 1 illustrates the percentage of students that exhibited each elective pattern, first in aggregate, and secondly only the students who targeted high-demand/low-supply disciplines. For the former, slightly over half of the class (52% percent) elected a High Dive pattern. The percentage of students that showed a Parallel Plan(s) pattern was slightly lower at 42%, and only 6% of the class exhibited a No Clear Pattern elective strategy. These percentages were similar for the students targeting HDLS disciplines (52%, 39% and 9% respectively).

**Table 1:** Percentage of 2017/2018 students that adopted each of the three main elective patterns in aggregate (row 1) and of those who targeted high demand/ low supply disciplines (row 2)

	High Dive	Parallel Plan(s)	No Clear Pattern
**All students who entered the CaRMS match**	52.1% (283/543)	41.8% (227/543)	6.1% (33/543)
**Students who targeted High demand/Low supply disciplines**	52.4% (111/212)	39.2% (83/212)	8.5% (18/212)

### Pre-CaRMS electives and match outcome

**Figure 1:**
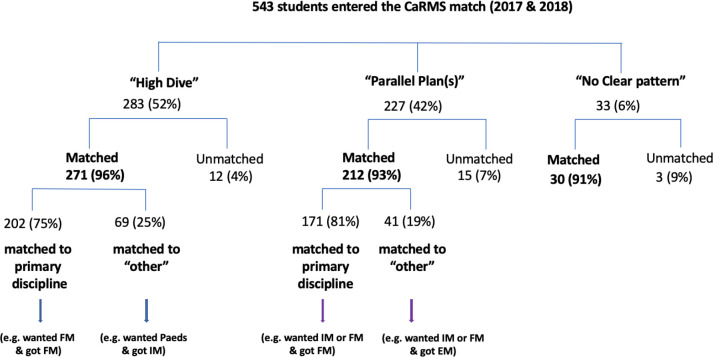
Pre-CaRMS elective patterns for the classes of 2017 and 2018

We show the relationships between each of the pre-CaRMS elective patterns and match outcomes in Figure 1.

The match rates for High Dive, Parallel Plan(s) and No Clear Pattern groups were not statistically different: 96% (95% CI = 93.4-98.1%), 93% (90.2-96.6%) and 91% (81.1-100%), respectively; χ^2^ = 2.2, *p* > 0.3.

While the match rate was 96% for the High Dive category, it is important to note that among the students who matched, 25% (95%CI = 20.3-30.6%) did so to a discipline other than what we inferred to be their primary discipline. We called this “matched to other.” An example of this is a student with a pattern of 10 or more weeks of Internal Medicine who matched to Family Medicine.

This was also true for students who exhibited the Parallel Plan(s) pattern, where 19% (14.9-24.7%) of the students matched to a specialty other than what we inferred to be one of their primary disciplines. Similarly, we termed this “matched to other.” An example of this is a student with six weeks of electives in both Internal Medicine and Family Medicine who matched to Radiation Oncology. The difference between the rate at which matched students matched to their primary discipline across elective patterns was not statistically different; χ^2^ = 2.5, *p* > 0.1.

**Figure 2:**
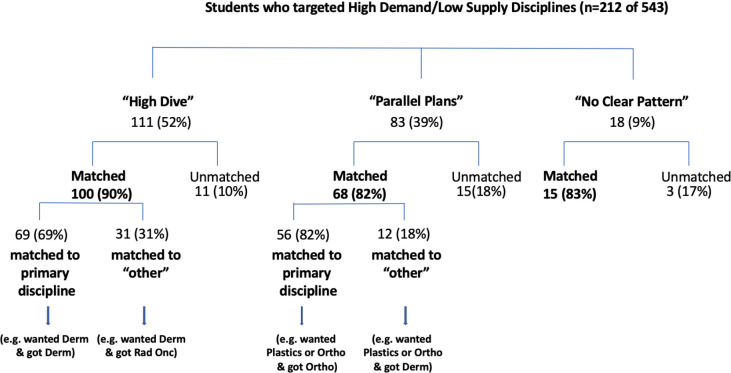
Pre-CaRMS elective categories containing only high-demand/low-supply disciplines (that is, Internal Medicine and Family Medicine removed)

We reviewed the specific disciplines that were pursued by students who were using the High Dive approach. We do not show the specific programs to protect students’ identities. The majority of these High Dive students who matched successfully, did so to large-capacity disciplines such as Internal Medicine and Family Medicine. These numbers would overshadow the numbers about which we would be more concerned: that is students with High Dive patterns in HDLS disciplines. In this context, high-demand means high number of applicants historically and low-supply means there are few spots available historically. To address this, we removed the students who had the majority of their electives in Internal Medicine and Family Medicine from the subsequent calculations (Figure 2). Here we show the match outcomes with only high-demand/low-supply disciplines that included: Dermatology, Plastic Surgery, Emergency Medicine, Neurosurgery, Urology, Obstetrics & Gynecology, Ophthalmology, Otolaryngology, Neurology, Anesthesiology, Pediatrics, General Surgery, Cardiac Surgery, Diagnostic Radiology, Orthopedic Surgery, Anatomical Pathology, General Pathology, Haematological Pathology, Medical Microbiology, Public Health & Preventative Medicine, Radiation Oncology, and Physical Medicine & Rehabilitation. The percentage of students who had the High Dive pattern of electives and who applied to high-demand/low-supply disciplines, was 52.4%. The percentage of match success remained high, at 90% (95% CI = 84.5-95.6%). This proportion of success matching was not statistically different from the rate at which both Parallel Plan or No Clear Plan students who prioritized HDLS specialties matched successfully (81.9% 95% CI = 73.6-90.2% and 83.3% 95% CI = 66.1-100%, respectively; χ^2^ = 2.8, *p* > 0.2). Furthermore, the majority of students with the High Dive pattern did match to the high-demand/low-supply specialties for which we assume they were aiming, at 69% (59.9-78.1%). Again, this proportion was not statistically different from the rate at which Parallel Plan students who, after prioritizing HDLS specialties, matched to one of the specialties for which we assume they were aiming (82.4%; 95% CI = 73.3-91.4% χ^2^ = 3.8, *p* = 0.05). However, a surprising 31% (21.9-40.1%) of the students who matched from the High Dive pattern did so to a discipline other than that in which they had the majority of their electives, including HDLS disciplines such as Plastics, Dermatology, and Anesthesiology.

### Students who did not match

There were unmatched students in each of the three electives patterns. The majority of the High Dive students who went unmatched did a High Dive for a high-demand/low-supply discipline. The majority of the Parallel Plan(s) students who went unmatched also included at least one (if not two) high-demand/low-supply discipline in their elective patterns.

## Discussion

We classified pre-CaRMS electives for UBC UGME senior medical students into one of three patterns: High Dive, Parallel Plan(s), and No Clear Pattern. Approximately the same percentage of students showed the High Dive and Parallel Plan(s) patterns with the overall match success being very similar for both patterns.

We did not expect this findingWe had underestimated the number of students that elected to employ a High Dive strategy. As well, we had made the assumption that only the students who were hoping to match to high-demand/low-supply disciplines would pursue such an approach. Secondly, we found a higher match rate for students with a High Dive pattern than we anticipated, including to disciplines other than the ones in which the students had completed 10 or more weeks of electives. Our interpretation is that the High Dive elective strategy could be successful but not necessarily to the primary discipline in which the electives were done. We propose two reasons for the high match rate using the High Dive approach: firstly, many students seemed to choose a High Dive strategy for large-capacity disciplines (Family Medicine and Internal Medicine); Secondly, a moderate percentage of the matched students in the High Dive category actually matched to a discipline that was not the one in which they had done the High Dive (an example: majority of Plastic Surgery electives and matched to Ortho). This meant that, despite having a predominance of electives in another discipline, these students were able to obtain interviews and then successfully match to another discipline in which they had done far fewer weeks of electives.

Similarly, although the majority of students who divided their electives between two disciplines in the Parallel Plan(s) category matched to one of these primary disciplines, students did also match to other disciplines, most to Family Medicine. Interestingly, however, some students matched to high-demand/low-supply disciplines, in the absence of many, or in some cases, any, pre-CaRMS electives in that discipline (an example: Parallel Plan(s) pattern for General Surgery and Plastics but matched to Dermatology).

We propose that these observations mean a viable back-up plan may reside in the application as a whole, rather than solely in the elective selection process. Future studies could include an exploration into alternative explanations (aside from electives). Did students include post-CaRMS electives or non-curricular clinical activities, which they described in their personal letters or interviews? Alternatively did they develop a cohesive and believable story about their career choice planning and elective selection? Finally did they apply broadly, and/or did they submit very broad and long rank order lists? Another important area for future research would be whether students could not change their electives even if their career choice changed and that could explain matching to a discipline other than that for which they appeared to be high diving. Faculty doing career advising should therefore pay close attention to students’ entire application**s**, and not only to their elective choices.

The observation that students restrict the breadth of their pre-CaRMS electives to increase their chances of matching to a particular discipline is consistent with studies that have examined the value students place on their senior medical student electives. In particular, applicants to surgical specialties agreed that the purpose of completing senior medical student electives was to maximize their match likelihood.^7^ In a mixed methods study, senior medical student students prioritized electives that related to “career development/preparation” (e.g., electives that allowed them to obtain letters of recommendation to support their match application).^8^ Orthopedic Surgery program applicants rated “desire to match” as the most important factor for arranging electives in their desired discipline.^9^ Given the current residency landscape, with more students going unmatched each year, students will feel they need to employ whatever strategy might increase their likelihood of matching, including narrowing the breadth of their electives.

We also did not expect to find that even the students who appeared to have no discernable pattern to their pre-CaRMS elective choices (No Clear Pattern) also achieved a high match rate. A handful of students with No Clear Pattern also matched to HDLS disciplines. We interpret that these students may have adopted a truly generalist approach to their senior medical student electives and that, if they presented this in a compelling way, they had a successful strategy to receive interview offers and ultimately to match. Consistent with this hypothesis, some program directors advocated for broader experiences rather than career-specific electives for students.^7^ The students in our study who elected a No Clear Pattern probably matched as a result of all the components of their application strategy: personal letters, curriculum vitae, references, research experience, interview preparation, and rank order list strategy.

Interestingly, the percentage of students who went unmatched was roughly similar for students whose elective patterns were classified into a High Dive, Parallel Plan(s), or No Clear Pattern; all strategies yield a relatively similar risk of going unmatched. Most students who went unmatched in each year did so having apparently aimed for high-demand/low-supply disciplines. This observation is consistent with data from unmatched students in the US system.^10^ Many of us have had this information provided to us anecdotally from our unmatched students over many years, and have not been aware of the number of students who actually do match with a High Dive pattern. We have believed that going “all-in” was a recipe for not matching.

Despite a 2018 report by the Association of Faculties of Medicine of Canada (AFMC) entitled: Reducing the Number of Unmatched Canadian Graduates, asserting that “Electives are intended to provide students with a means to address self-identified or formally specified weaknesses, explore a variety of clinical practice environments and disciplines, and achieve breadth of scope in knowledge, skills and attitudes,” over half of the students in our two-year study cohort chose a pattern of electives that seemed directed at increasing their match likelihood to one (or possibly two) disciplines.^11^ The AFMC report also suggested that students who prioritize pre-CaRMS electives in high-demand/low-supply disciplines might disadvantage themselves if they do not match to the specific discipline for which they were aiming because they then are not seen as competitive in other disciplines. We did not find this to be completely true for first-iteration applications to other disciplines. It certainly might be true for second-iteration applications. The students in our study who elected High Dive patterns had a similar overall percentage match rate to the whole class and also did match to disciplines other than those in which they had done most of their electives. We suggest, from our data, that students employing High Dive strategies may not compromise their first iteration competitiveness as much as we had originally thought. To this end, we would like to stress that elective strategy is only one of several important contributors to match success.

### Limitations

We have identified the following limitations to this study:

- Our definition of “match success” may not represent what students consider as success since we did not have access to their personal match planning or their rank order lists.
- We inferred students’ primary discipline choice(s) only through reviewing their combination of electives and thus cannot infer causation from correlations. A student may have had a High Dive pattern of Plastics electives but matched to Internal Medicine because they decided at a later point to prioritize Internal Medicine over Plastics.
- We did not make a distinction between in-province and out-of-province electives, nor did we track locations or supervisors within UBC, or the details of the electives themselves.
- We included but did not distinguish data from students who entered the CaRMS match as couples. That said, fewer than five couples (over both years) were included thus we do not believe this substantially influenced the conclusions.
- Comparatively small numbers of students successfully matched to the high-demand/low-supply disciplines and thus we are limited in our inferences about this group. We were even more limited in drawing inferences about specific disciplines, such as Plastic Surgery, Otolaryngology, or Dermatology.
- Finally, we examined two years at a single medical school, and one with more pre-CaRMS elective time than other schools in Canada. Our findings may not be generalizable to all Canadian medical schools.

## Conclusion

Students with High Dive and Parallel Plan(s) patterns of pre-CaRMS electives at UBC matched with similarly high rates with relatively low numbers of students not matching. Most students with these patterns matched primarily to the high-capacity disciplines of Family Medicine and Internal Medicine. Students with High Dive patterns with high-demand/low-supply disciplines matched to what we inferred were their goal disciplines at 69%. The rest of these students who matched, did so to a discipline that had not been a major part of the students’ elective patterns.

Elective selection alone, while contributory, is not the only component to first iteration match success. Having a viable back-up plan may reside in the application as a whole, rather than solely in the elective selected. An avenue for future research could include the relationship between the student’s ability to convey a cohesive story in their personal letters that supports more than one discipline choice and the likelihood of matching. We should also encourage students to invest in other aspects of their application package such as the content of their personal letters, curriculum vitae, research, reference letters, and interview practice.
